# Solid Stress-Distribution-Oriented Design and Topology Optimization of 3D-Printed Heterogeneous Lattice Structures with Light Weight and High Specific Rigidity

**DOI:** 10.3390/polym14142807

**Published:** 2022-07-09

**Authors:** Bo Li, Ciming Shen

**Affiliations:** 1 School of Mechanical and Power Engineering, East China University of Science and Technology, Shanghai 200237, China; scm1997ant@163.com; 2 Shanghai Collaborative Innovation Center for High-End Equipment Reliability, Shanghai 200237, China

**Keywords:** heterogeneous lattice, lightweight, topology optimization, 3D printing, structural design, resin

## Abstract

Lightweight structural design is greatly valued in the aviation, aerospace, and automotive industries. Three-dimensional (3D) printing techniques provide viable and popular technical pathways for the rapid design and manufacturing of lightweight lattice structures. Unlike the conventional design idea of a geometrically homogenized lattice structure, this work provides a design method for structurally heterogeneous lattice according to the spatial stress state of 3D-printed parts. Following the quasi-static stress numerical simulations of solid components, finite element mesh units were inconsistently replaced by lattice units with different specific rigidities corresponding to the localized stress levels. Relying on the topology optimization further lightened the lattice structure under quasi-static stress after removing some parts with extremely low stress from the overall structure. As an embodiment of this design idea, face-centered cubic (FCC) lattice units with different strut diameters were employed to non-uniformly and adaptively fill a solid part under localized loading. The topological optimization was conducted on the solid part globally. Then, the topologically optimized solid and the heterogeneous lattice structure were subjected to the geometric Boolean operation. Stereolithographic 3D printing was utilized to fabricate the homogeneous and heterogeneous lattice structural parts for comparative tests of three-point bending. Three evaluation indicators were defined for the standardized assessment of the geometrically complex lattice structures for the performance evaluation. This demonstrated that the heterogeneous lattice part exhibited better comprehensive mechanical performance than the uniform lattice. This work proved the feasibility of this new perspective on 3D-printed lightweight structure design and topology optimization.

## 1. Introduction

The additive manufacturing (AM) technique, also popularly called 3D printing, exhibits remarkable technical advantages for the rapid and free design and fabrication of geometrically complex parts, compared with conventional manufacturing processes [1,2]. Customized lightweight parts of various metals and/or non-metallic materials can be 3D printed via multiple AM methods, such as powder bed fusion (PBF), stereolithography apparatus (SLA), fused deposition modeling (FDM), etc., following the computer-aided design (CAD) of digital models [3]. AM has developed rapidly in many industrial fields [4]. Three-dimensional (3D) printing is always desirably employed to fabricate lattice structural components with lightweight targets [4,5,6]. Lattice structures with complex configurations constantly receive attention due to their performance advantages, including high specific stiffness or specific strength and promoted energy absorption capacity [7,8].

Various lattice structural components can be artificially created and geometrically optimized through CAD. Each lattice unit of the periodically arrayed lattice structure comprises struts and nodes interconnected in 3D space [9]. The mechanical properties of each lattice unit with the determined shape dimensions are related to the lattice-unit geometry, strut diameter, number of nodes, and geometries of the struts and nodes. As the numbers of struts and nodes per unit of space volume in the lattice structure increase, the stress distribution is more dispersed. This leads to a higher strength or stiffness of the whole lattice structure. However, the density or mass per unit of volume of the overall lattice part increases, in contrast to the light weight goals. Fabricating highly dense lattice structures also presents challenges [10,11,12]. Conversely, suppose that the distribution of struts and nodes in the lattice structure is too sparse. In that case, it is not conducive to guaranteeing the mechanical strength or stiffness of the overall lattice structure. Therefore, establishing a trade-off between light weight and overall structural strength or stiffness is the dominant idea for engineering design methods for lattice structures.

In recent years, many studies on designing lightweight lattice structures have been reported. For instance, the voxel-based lattice design method promotes computational efficiency and geometric design flexibility [11]. Wang et al. used a multiscale parallel design method to simultaneously optimize the macro- and microstructures at different scales [13]. Some researchers have performed several size-adaptive matching and scale-adjustable design methods according to the design variables of lattice-unit cross-sectional geometry and dimensions [14,15]. Stefan et al. studied the optimization of lattice structural struts arranged according to the applied loading direction, aiming to give full play to the stress-bearing capacity of each strut in the entire structure [16,17]. Jin et al. proposed a global–local design approach for the gradient lattice structures [12]. Different lattice-unit types were chosen for different regions in the lattice structure [12]. Moreover, the diameters of the struts were optimized to achieve a higher specific strength of the entire structure, considering the applied stress conditions [12]. Kang et al. deduced the criteria for the mechanical performance of lattice structures based on the relative densities of different types of lattice units [18]. Pham et al. claimed that the stress of the lattice unit has a distinct orientation trait, guiding people to design an anisotropic lattice structure that meets the actual working needs [19]. To further improve the comprehensive performance of lattice structural parts, researchers have also advocated using topology optimization to model the lattice structures with different densities [20,21]. For instance, Chen et al. proposed a dimension optimization algorithm based on the moving isosurface threshold (MIST) to topologically optimize the lattice structures and improve the mechanical properties per unit of mass [22]. Dong et al. introduced a model of hybrid lattice units to simulate the lattice mechanical properties, thereby optimizing the design of heterogeneous lattice-unit distributions in the lattice structures [23]. They verified the design theory and superiority of this hybrid multimode lattice structure with a three-point bending beam [23]. Teimouri et al. assembled a topology-optimized solid structure from various lattice structures obtained via a method based on bidirectional evolutionary structure optimization (BESO) [24]. Accordingly, they built a new hybrid solid–lattice structure, and demonstrated the superiority of the structure through stiffness, modal, and quasi-static finite element analysis (FEA) [24].

To sum up, it can be inferred that various promising design methods breaking the stereotyped ideas are still needed for 3D-printed lattice structural innovation. At present, the researchers engaged in lattice structure design have built a consensus on how to achieve a high specific strength or specific stiffness of lattice structural parts or conduct the lightweight limit design. It is inferior to rely only on uniform lattice-unit arrays to surpass the non-uniformly distributed lattice structures. However, there is still no simple, easy-to-engineer, and generally accepted design approach for heterogeneous lattice structures with non-uniform or variable density distributions of various lattice-unit types. Moreover, researchers have developed some characterization and evaluation methods for stress, strain, and buckling behavior to obtain more detailed performance information of lattice structures in terms of stiffness, strength, deformation, impact energy absorption, etc. These assessment methods are the prerequisite research to improve the lattice structural design method.

In this work, the aim was to fully exploit the technical advantages of 3D printing to further develop the design concept of lattice structures—especially the simple design approach that is generally accepted and applied to engineering applications. Herein, we studied a structurally heterogeneous lattice design method suitable for the quasi-static stress state of 3D-printed parts. Based on the quasi-static stress numerical simulation of the solid part, the finite element (FE) mesh units were replaced by different lattice units suitable for the localized stress levels. As a concrete example, face-centered cubic (FCC) lattice units with varying strut diameters were employed to non-uniformly and adaptively fill a solid bar under localized loading. Furthermore, a topology optimization approach was employed for further lightening the heterogeneous lattice structure by removing some parts—which suffered extremely low stresses—from the entire structure. Then, the topologically optimized solid and the designed heterogeneous lattice structure were merged after being subjecting to the geometric Boolean operation. SLA 3D printing was utilized to fabricate homogeneous and heterogeneous lattice structural parts for the comparative tests and performance evaluation of three-point bending. Some evaluation indicators suitable for the standardized assessment of heterogeneous lattice structures were further determined. This work proves the feasibility of a heterogeneous structural design perspective on 3D-printed lattice structures with topology optimization.

In general, the lightweight method practiced in this work is simple and suitable for engineering applications. We consider the non-uniform lattice after the replacement design based on the discretization of its spatial stress or strain field distribution, along with the further lightweight by removing unnecessary localized parts. The difference in lattice rod diameter in different local structures can be designed for continuous rod-diameter changes. This reduces the stress concentration of new non-uniform lattice parts. Under the known static or quasi-static load-bearing conditions of the structural parts, redundant localized parts with no or little load can be directly removed to facilitate lighter weight. Although this work only uses a simple cuboid structure and a simple three-point bending case to verify and illustrate the design method, the idea of a relatively simple replacement–removal path employed in the lightweight engineering of static load-bearing structures with complex geometries is promising.

## 2. Materials and Methods

### 2.1. General Design Approach for Heterogeneous Lattice Structures

**Figure 1:**
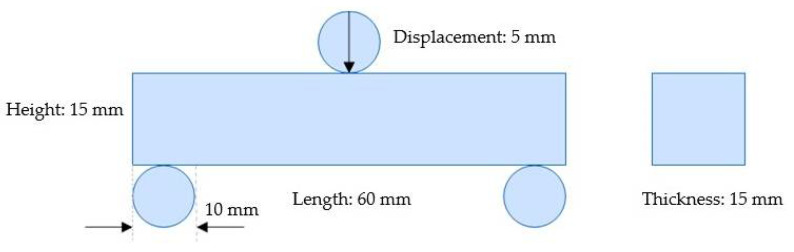
Geometric boundary conditions of the verification part for FEA.

**Figure 2:**
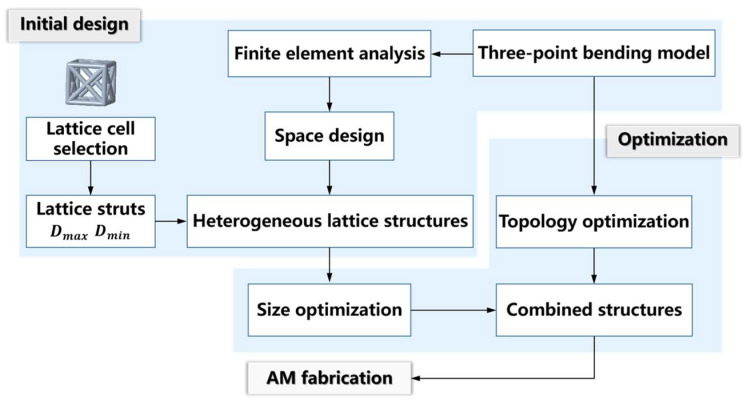
The design flow of heterogeneous lattice structures in this work.

The conventional method for designing lattice structures is to populate solid parts with lattice units of a single geometric form. Although the 3D modeling process of the homogeneous lattice-unit-filled structure is simple, it is hard for the as-built lattice structure to achieve the light weight limit that is adapted to the service status. In contrast, the 3D modeling of the heterogeneously hybrid lattice structures is more challenging. In this work, FCC lattice units with different strut diameters were arrayed to fill a solid bar under the localized three-point bending load. The dimensions of the bar used in this work, suffering three-point bending, are shown in Figure 1, which also exhibits the geometric boundary conditions for the applied loads. Short beams generally refer to beams whose span-to-height ratio is 2~5. The length and height of the beam sample in this work were 70 mm and 15 mm, respectively, meeting the requirements of short beams. We generally divide the design path of heterogeneous lattice structures into two stages, as indicated in Figure 2.

Initial design stage: Stress–strain FEA that meets service requirements was performed on the solid part, as the lattice design object, to determine the low-stress and high-stress regions. The bar suffered quasi-static three-point bending stress within a certain range, guiding the FEA modeling and simulated stress–strain calculation. According to the stress-filed nephogram by FEA, the local regions with different stress degrees were filled with lattice units with various stiffness characteristics. In this work, we replaced the solid regions with different FCC lattice units with three strut diameters to initially generate a lightweight lattice structure.

Optimization design stage: Modifying connection geometries between the adjacent lattice units, conducting the necessary geometric adjustment of the lattice units themselves, implementing topology optimization of the entire structure’s external shape for further weight reduction, and generating a hybrid heterogeneous lattice structure through a geometric Boolean operation of the topologically optimized outer shape and the heterogeneous lattice structure.

### 2.2. Material, Fabrication, and Three-Point Bending Testing of Lattice Structural Samples

This work used SLA 3D printing to fabricate lattice structures for three-point bending tests, aiming to verify the effectiveness of the structural design. The SLA equipment used to manufacture samples was Formlabs Form3^TM^. The 3D-printed samples included the heterogeneous lattice structure, according to the design results, and the homogenized lattice structure as a control sample.

The raw material used in the SLA 3D printing fabrication was Grey Photopolymer Resin^TM^ from Formlabs. After post-curing treatment, the ultimate tensile strength, tensile modulus, elongation, flexural stress at 5% strain, flexural modulus, and notched Izod impact strength were 61 MPa, 2.6 GPa, 13%, 86 MPa, 2.2 GPa, and 18.7 J/m, respectively. The density and Poisson’s ratio of this post-cured resin were 1.12 g/cm^3^ and 0.23, respectively.

The laser spot diameter of SLA printing was 85 μm. We set the print layer thickness to 25 μm. A cross-scanning strategy with an SLA device at a default scan rate of 3 m/s ensured that the resin was printed with high density. In addition, uncured resin slurry was attached to the surface of the as-printed samples. Hence, the samples were placed in an ethanol solution and cleaned in an ultrasonic cleaner for 3 min. The ethanol solution dissolved the resin slurry adhering to the sample surface. The cleaning duration needed to be optimized experimentally. If the cleaning time was too short, there would still be residual liquid resin. Conversely, when the cleaning time was too long, ethanol would dissolve part of the cured resin, resulting in surface defects of the samples. After cleaning the as-printed samples, an air-dryer quickly evaporated the ethanol on the sample surface. After that, the samples were intensively cured for 3 min in a UV-lamp curing equipment, and then the post-processing was completed.

Three-point bending mechanical performance tests on the heterogeneous lattice samples and the control samples with homogeneous lattice units were performed using a universal mechanical performance test machine. In the three-point bending testing, the sample was placed in a set position between two points. At a certain position in the middle of the two points, the sample was loaded vertically downward at a certain rate. The sample was bent until it was broken. The applied loading rate was a constant value of 0.5 mm/min. The load–displacement curves of the samples were plotted.

## 3. Initial Design of a Stress-Adapted Heterogeneous Lattice Structure

### 3.1. Mechanical Performance-Guided Lightweight Design Target

In this study, the primary design target was promoting the entire lattice’s specific stiffness and light weight. As a representation of the ease of elastic deformation, the stiffness of a lattice structure refers to its ability to resist the whole structural elastic deformation when subjected to the applied force [25]. This determines the lattice’s structural stability, and governs its micro-deformation degree [26]. Nevertheless, a lattice structure with high stiffness tends to have relatively poor impact resistance or low energy-absorption capacity. Thus, evaluating the comprehensive performance of lattice structures should also consider the mechanical indices during cracking failure behavior. Accordingly, for a typical illustration, we designed, tested, analyzed, and verified the heterogeneous lattice structure’s comprehensive mechanical properties based on the three-point bending stress condition. The deformation amount and failure morphology of different lattice structures at the moment of fracture can be comprehensively examined considering the bending stiffness, brittle failure risk, and light weight, so that the differences between various types of lattice structures can be carefully compared.

The choice of the geometric type of lattice units is one of the most critical parts of the design procedure of lattice structures. Countless geometric types of lattice units can be chosen, including the commonly used FCC unit, body-centered cubic (BCC) unit, gyroid unit, etc. Different lattice-unit types exhibit different mechanical properties, including stiffness, strength, coordinated deformation capacity, and energy-absorption capacity. Of course, the lattice parent material properties and manufacturing process parameters also affect their mechanics. For lattices composed of microscale struts and node connections, the structural stability of the lattice unit can be analyzed according to the Maxwell stability criterion equation, as follows [27]:

(1) M=n−3j+6

where *n* is the number of struts, *j* is the number of nodes, and *M* is the Maxwell stability coefficient representing the structural stability. If the *M* of the lattice-unit structure is less than zero, the structure is classified as preferentially bending. Otherwise, it is classified as preferentially stretching [27]. For example, if the *M* of an FCC lattice unit is −12, i.e., *M_FCC_* = −12, it is classified as a bending-dominant lattice.

When a bending-dominant lattice unit is subjected to applied force, the force on the strut is similar to that of a curved beam. Its deformation depends on structural parameters such as the strut diameter, appearance frame dimensions of the lattice unit, etc. This work mainly used FCC lattice units with different strut diameters and external sizes to design heterogeneous lattice structures.

### 3.2. Stress-Adapted Arrangements of Different-Sized Lattice Units

**Figure 3:**
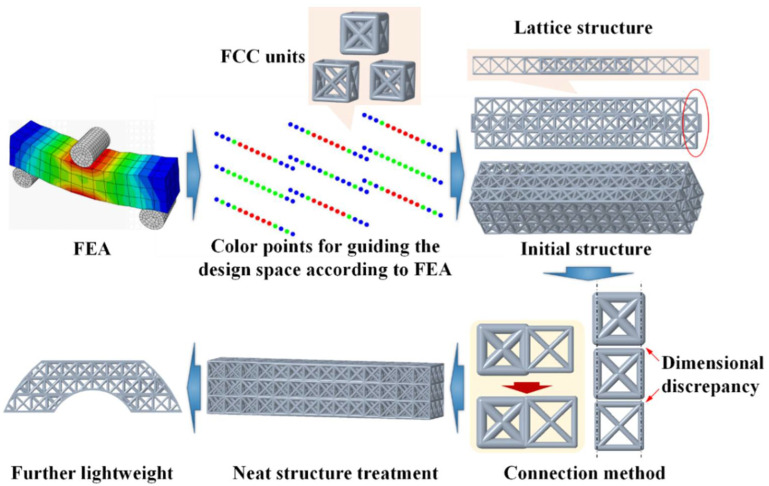
The initial design of the hybrid structure.

The 3D nephogram of stress distribution in the solid structure was built from the FEA of the three-point bending. The equivalent stress data of the structure were divided into three intervals of [0, 200), [200, 300], and [300, +], which were separately represented in blue, green, and red in a new “pseudo-stress-distribution nephogram” containing only the three cubic stress meshes. Then, the geometric centers of these three types of cubic meshes were converted into the corresponding tricolor point map. The distribution space of the point map was defined as the design space, as indicated in Figure 3. Considering the accuracy and manufacturability of SLA 3D printing, three strut diameters (0.6 mm, 0.8 mm, and 1 mm) were tailored for the FCC lattice units. The design area was filled with these three types of FCC lattice units, according to the following rules:

(1) The higher equivalent stress region was filled using the FCC lattice units with larger strut diameters.

(2) The connection between the adjacent lattice units with different strut diameters needed to be adjusted according to the outline frames of the lattice units, so that the side struts of the adjacent lattice-unit outline frames were overlapped, as shown in Figure 3.

The initial design structure of the heterogeneous lattices was modeled by CAD, as shown in Figure 3. After the combination of lattice units with different strut diameters due to the dimensional discrepancy of the side struts, the geometry of the entire lattice structure differed from that of the original solid structure (Figure 3). Hence, it required a further optimization design stage.

## 4. Optimization Design of a Further-Lightened Heterogeneous Lattice Structure

### 4.1. Dimensional Optimization of Lattice Units

**Figure 4:**
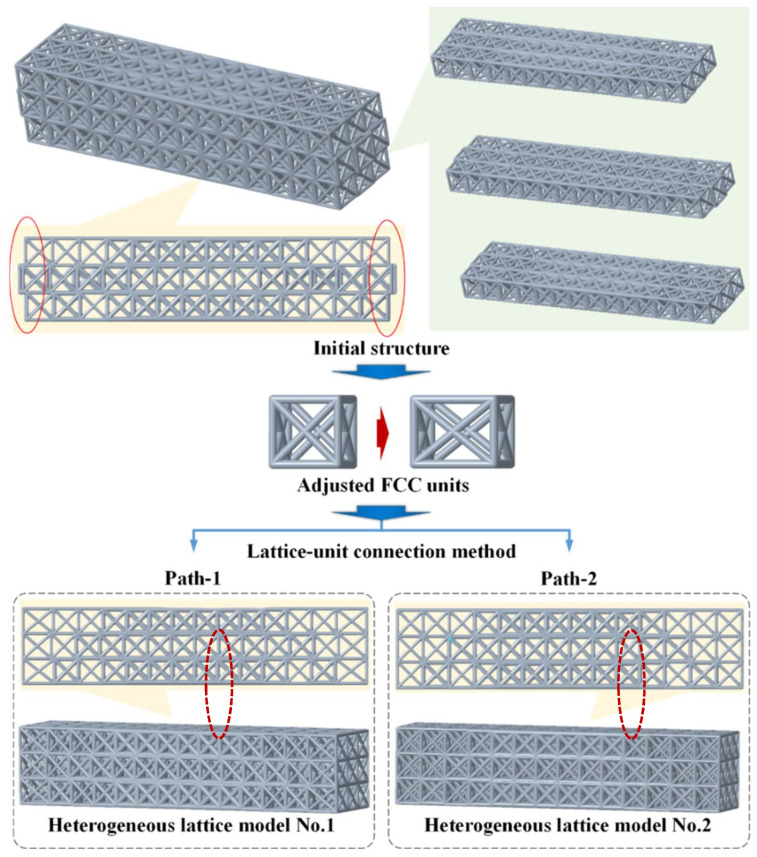
The design flow of the two structural optimization paths.

Aiming to solve the abovementioned lattice-unit frame dimensional discrepancy issue, two processing paths to achieve the consistency of lattice-unit frame connection dimensions were conducted. The design process is shown in Figure 4.

In processing path 1, only the lattice units at the frame-side locations of the design regions were replaced with FCC lattice units with altered sizes (Figure 4). Although this method ensured that the entire geometric shape of the heterogeneous lattice structure was consistent with the original solid structure, it caused the geometric distortion of the lattice units at the frame locations to be too large. This led to a decrease in stiffness in the boundary regions of the lattice structural frames. In addition, if the length of the horizontal struts in the lattice unit was too long, the horizontal struts would sag or even fail to form during the 3D-printing process.

In processing path 2, we generated all vertical struts distributed on the aligned verticals for all of the lattice units of a lattice structure, as shown in Figure 4. This ensured the load-bearing capacity of the struts in the vertical direction of the heterogeneous lattice structure. However, it changed the sizes of almost all of the FCC lattice units. We called this design idea the self-adaptive design of lattices; that is, it adapted not only to the structural stress distributions, but also to the overall shape and the collinear geometry of the struts in the primary load-bearing direction (Figure 4).

### 4.2. Topology Optimization for the Further Weight Reduction

**Figure 5:**
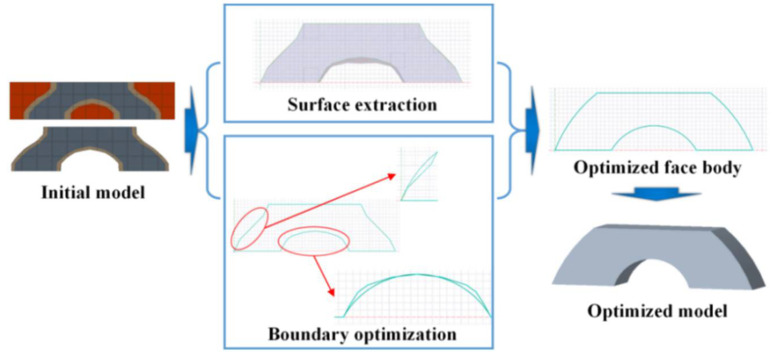
The schematic of the generation process of the topology-optimized model for further solid-body weight reduction under the load-bearing orientation and stress distribution.

The topology optimization improved the entire structural stiffness and reduced the weight of the heterogeneous lattice structure in this work. Although there have been some reports on the geometric optimization design of lattice structures subjected to three-point bending, most focused on improving the lattice structures themselves. They did not try to remove redundant structures on the whole to achieve further weight reduction [28,29]. We used the solid isotropic material with penalization (SIMP) algorithm to optimize the design space. The topology optimization goals determined the structural stiffness maximization and the apparent volume minimization. The FE simulation method was employed to gradually add voxels into the design domain or delete them from it. The iterative calculations were performed until the objective function converged and the volume achieved the constraint value. The mathematical expressions for this method were as follows (Figure 5):

(2) $$\mathrm{Objective}:\;f\left(\rho ,U\right)=U^{T}KU$$

(3) Subject: K(ρ)U=F(ρ)

(4) $$V^{*}=\sum_{i=1}^{n}v_{i}\rho^{*}$$

(5) $$0\leq \rho_{\min}\leq \rho^{*}\leq \rho_{\max}\leq 1$$

where the objective function *f* in Equation (2) is the structural compliance, *K* is the global stiffness matrix, *F* is the external load vector, and *U* is the displacement vector. The first constraint equation, Equation (3), is an equilibrium equation on solid structural topology optimization. The second constraint equation, Equation (4), refers to the limits of the entire designed structural volume, $V^{*}$. The volume constraint target was 50% weight reduction of the solid structure in this work. The third constraint equation, Equation (5), concerns the 3D printing manufacturability, thereby constraining the minimum and maximum values of the relative density of the lattice structure; $\rho^{*}$ in Equation (5) represents the relative density variable of each lattice structural voxel for the optimization algorithm. Fifteen iterative calculations were performed in this work, resulting in a 40% reduction in the total mass of the structure.

The preliminary topology optimization result of the as-marked “initial model” is shown in Figure 5. This initial model had rough boundaries. If the model was used directly, the SLA molding accuracy would be poor. Furthermore, a correction method was proposed to make the boundary surface smooth. The strategy included extracting the model, fitting the curve, and stretching the line body, as shown in Figure 5. The initial model was imported into the CAD software to obtain the bounding wireframe. Smooth curves were fitted to the boundary lines and then compared to the “initial model” wireframe. After each curve was optimized, the wireframe was extruded into a surface. Then, the plane was extruded into a body (Figure 5).

### 4.3. Geometric Boolean Operation for the Combination of Lattices and Topology-Optimized Outer Shape

**Figure 6:**
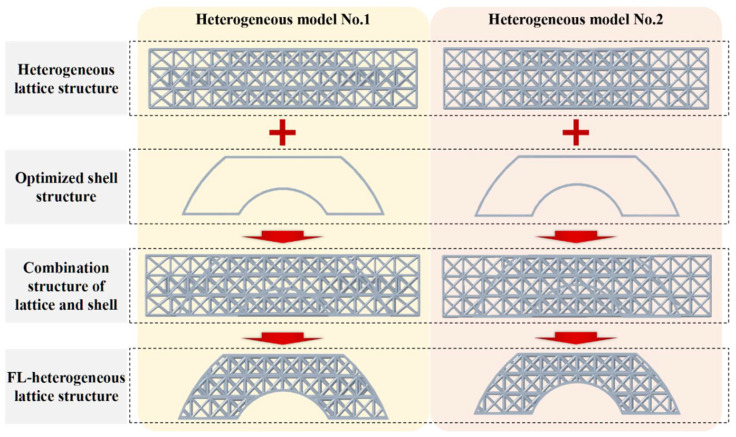
The schematic of the generation process of topology-optimized lattice structures for further weight reduction under the load-bearing orientation and stress distribution.

The designed heterogeneous lattice structures (Figure 4) and the shell structures further-lightened (FL-) by topology optimization (Figure 5) were combined by the geometric Boolean operation to construct the hybrid FL–heterogeneous lattice structure models, as exhibited in Figure 6. Moreover, the outer surface shell thickness of the FL–heterogeneous lattice structures was 0.6 mm. For further comparing the heterogeneous lattice structures, we constructed homogeneous structural models filled with FCC lattice units with strut diameters of 0.8 mm and 1 mm as the control samples for the three-point bending testing. Therefore, as shown in Table 1, eight lattice structures were modeled and 3D printed for comparative analyses.

Furthermore, it should be noted that we named the eight models as follows: (I) Heterogeneous lattice No. 1, (II) Heterogeneous lattice No. 2, (III) Uniform lattice No. 1, (IV) Uniform lattice No. 2, (V) FL–heterogeneous lattice No. 1, (VI) FL–heterogeneous lattice No. 2, (VII) FL–uniform lattice No. 1, and (VIII) FL–uniform lattice No. 2. The strut diameter was 0.8 mm in Model III and Model VII. The strut diameter was 1.0 mm in Model IV and Model VIII.

## 5. Experimental Results and Discussion

### 5.1. Mechanical Performance Indices for Comparisons

The lattice structures combined with the topology-optimized shell structure further lightened the entire component to be 3D printed. However, the differences in the specific stiffness and mechanical behavior of these structures still required further experimental verification. The stress-adapted arrangements of different-sized lattice units should be experimentally verified according to the original intention of increasing the specific stiffness of the lattice structures. To facilitate the comparative evaluation of lattice structure performance, we used the combination of relative density and flexural stiffness. The deflection of a three-point bending beam (simply supported beam) under loading can be expressed by Equation (6):

(6) $$\delta =\frac{PL^{3}}{48EI}$$

where the load, *P*, and the deflection, *δ*, can be obtained by the three-point bending test when the sample has a brittle failure. *EI* can be derived if the gage length and the flexural rigidity are known. In general, the equations of the relationship between the load and deflection of the sandwich panel can be transformed to obtain the equivalent bending stiffness and shear stiffness [30]. However, with the change in boundary conditions, the shape and geometric details of the lattice structure change. To intuitively compare and evaluate the designed lattice structures, we constructed new index parameters using Equation (6).

The relative bending stiffness and relative density can be expressed by Equation (7) and Equation (8), respectively. Then, the exponent in Equation (9) can be used to compare the flexural stiffness considering the relative density of the lattice structure.

(7) $$EI^{*}=\frac{EI_{L}}{EI_{S}}$$

(8) $$\rho^{*}=\frac{V_{L}}{V_{S}}$$

(9) $$\alpha =\frac{EI^{*}}{\rho^{*}}$$

where EI* is the relative flexural rigidity, *EI*_S_ is the flexural rigidity of the solid structure, *EI*_L_ is the flexural rigidity of the lattice structure, ρ* is the relative density of the lattice structure, *V*_s_ is the volume of the solid structure, *V*_L_ is the volume of the designed lattice structure, and *α* is the performance index of flexural rigidity with the degree of light weight. However, the *α* index cannot reflect the sample plasticity. Plasticity can also be manifested by deflection. A performance index *γ* was constructed using Equations (10) and (11) to comprehensively compare and evaluate the designed lattice structures; *γ* is the product of *α* and *β*, and is a comprehensive index considering plasticity and bending stiffness.

(10) $$\delta^{*}=\frac{\delta }{\delta_{S}}$$

(11) $$\beta =\frac{\delta^{*}}{\rho^{*}}$$

(12) γ=α∗β

where *δ*_S_ is the deflection of the solid structure when it experiences a brittle failure, δ* is the relative deflection, *β* is the performance index of plasticity with the degree of light weight, and *γ* is the performance index of the combination of flexural rigidity and plasticity.

### 5.2. Experimental Results

**Figure 7:**
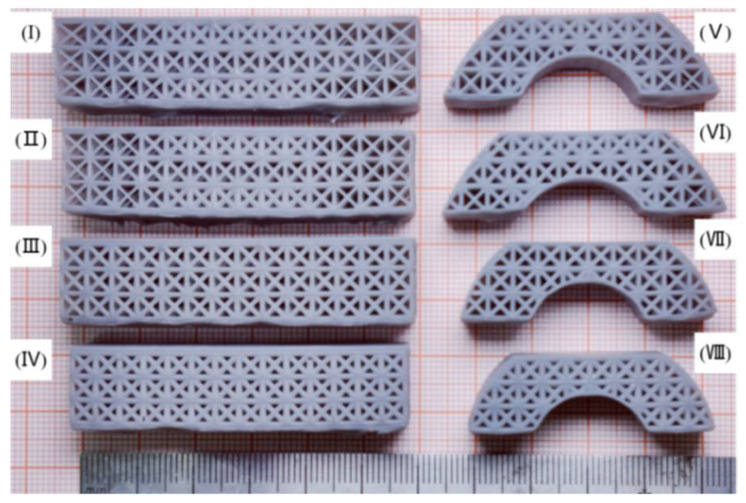
Eight types of lattice samples via SLA for three-point bending: (**I**) Heterogeneous lattice No. 1, (**II**) Heterogeneous lattice No. 2, (**III**) Uniform lattice No. 1, (**IV**) Uniform lattice No. 2, (**V**) FL–heterogeneous lattice No. 1, (**VI**) FL–heterogeneous lattice No. 2, (**VII**) FL–uniform lattice No. 1, (**VIII**) FL–uniform lattice No. 2.

**Figure 8:**
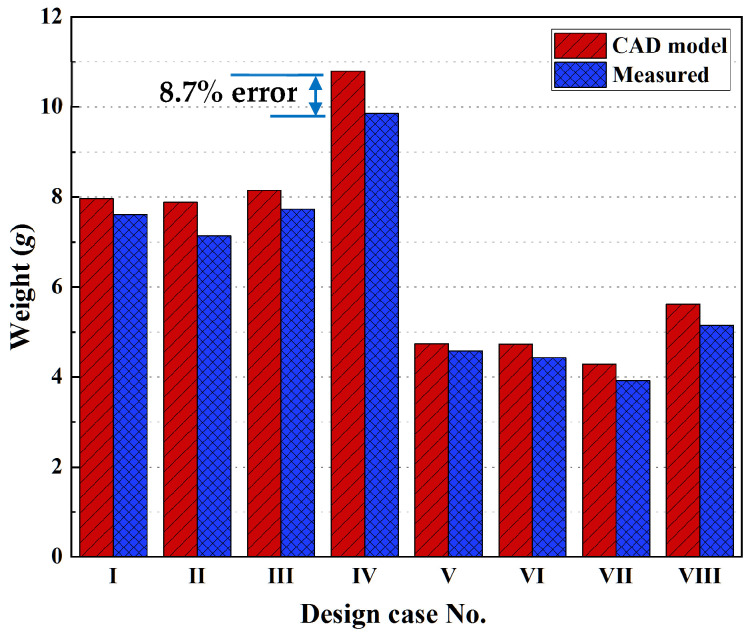
The weight of the eight types of lattice samples compared to their CAD models.

According to the eight designed models, the samples for three-point bending tests were fabricated by SLA 3D printing (Figure 7). The as-printed samples were measured to obtain their weight and compare it with the information from CAD models, as shown in Figure 8. It was confirmed that the dimensional accuracy of SLA 3D printing was relatively high, with a mass error of at least 3–10%. We inferred that the mass error was due to the alcohol employed to clean the uncured liquid resin, which dissolved the surface of the as-printed samples.

**Figure 9:**
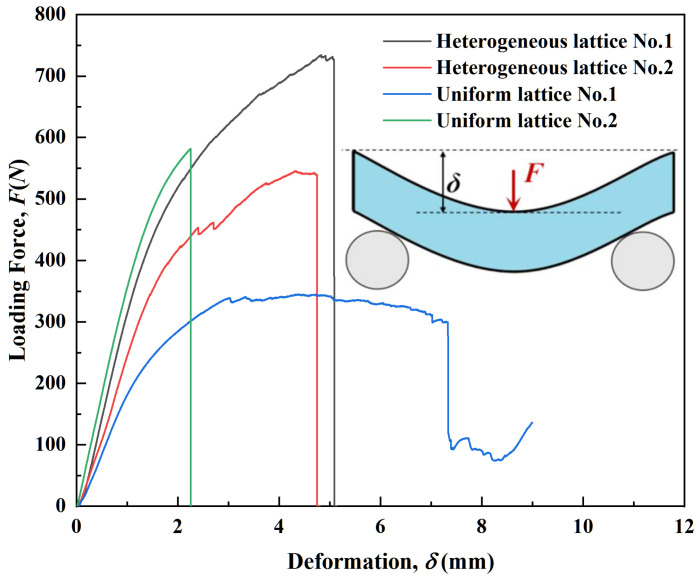
The bending load–displacement curves of the four lattice structures.

**Figure 10:**
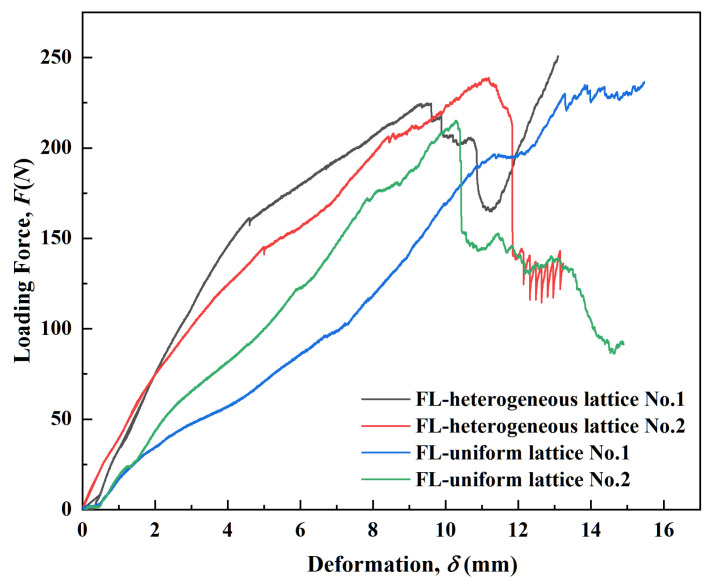
The bending load–displacement curves of the four lattice hybrid structures.

The load–displacement curves of the eight types of samples are shown in Figure 9 and Figure 10. The results implied that the mechanical properties of the lattice and topology-optimized hybrid structures were considerably different. The mechanical performance indices of the eight types of samples are compared and presented in Table 2. The four cuboid lattice structures showed greater stiffness in the initial deformation region than the topology-optimized ones (Figure 9 and Figure 10). The ultimate load (727.29 N) of the Model I sample was the largest of the four lattice structures. The ultimate load decreased in the hybrid and topology-optimized lattice structures compared to Model I, due to the low relative density of the topology-optimized lattice samples. The deflection calculation results showed different trends. The average plasticity of the four hybrid topology-optimized samples was higher. When there was apparent fracture failure in the four hybrid topology-optimized samples, the average position shift was more significant than that of the cuboid lattice structure samples without further lightweight topology optimization. The three-point bending load–displacement curve of Model VII differed from the others. It was considered that the deviation existed due to the 3D modeling error (Table 1 and Figure 7) compared to the actual shape, along with SLA-induced defects in the samples. In addition, the load–displacement curve of three-point bending on some lattice structures exhibited the characteristics of secondary strengthening. The second or multiple secondary strengthening peaks appeared after the first load peak in the curve. The literature also verified this phenomenon [28,29,31]. After local densification, it resulted from the local strengthening effect in the overall lattice structure. Meanwhile, the investigation of the mechanical properties of 3D-printed elastomers is different from that of vitrimers or 3D-printed metals [32,33]. In the following sections, we devote more attention to the analysis, evaluation, and deconstruction of the designed and 3D-printed lattice structural stiffness indices.

### 5.3. Mechanical Performance of Heterogeneous Lattices Compared to the Uniform Ones

By comparing the mechanical properties of the four heterogeneous lattice samples and the four uniform ones (Table 2), and employing the calculation result of the original solid structure as a reference, the relative bending stiffness and plastic properties were calculated according to Equations (9) and (11). The samples with the most prominent and most minor relative density values were Model IV ($\rho_{\mathrm{IV}}=0.582$) and Model VII ($\rho_{\mathrm{VII}}=0.231$), respectively. Compared to the uniform lattice samples, the index of relative flexural rigidity, α, showed that Model I and Model IV had maximum values of 0.341 and 0.347, respectively. The relative density of Model IV was greater than that of Model I. Moreover, in the topology-optimized structures, the FL–heterogeneous lattice No. 1 (Model IV) had a relative flexural rigidity of 0.037; it was also greater than that of the FL–uniform lattice samples. Then, the plasticity index, *β*, as the most considerable value, was 2.601 in the lattice structures (Model I). Models I and II had significantly higher plasticity than the other lattice structures in the experimental testing. Moreover, all of the topology-optimized structures had better plasticity. When the defined comprehensive index *γ* was derived according to Equation (12), the maximum value achieved was 0.886 in the heterogeneous lattice structure sample of Model I. Considering the lightweight design goal, this indicated that the heterogeneous lattices promoted the specific stiffness.

### 5.4. Macroscale Perspective of Failure Behavior

**Figure 11:**
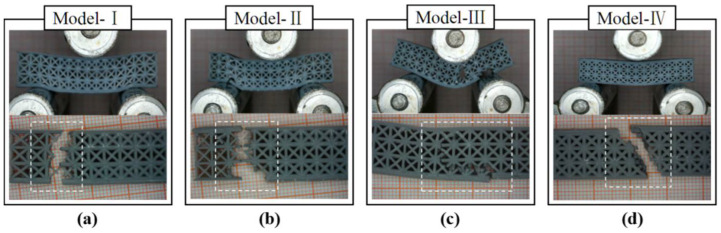
The failure modes of the proposed lattice structures: (**a**) Heterogeneous lattice No. 1, (**b**) Heterogeneous lattice No. 2, (**c**) Uniform model with FCC strut diameter of 0.8 mm, (**d**) Uniform model with FCC strut diameter of 1 mm.

**Figure 12:**
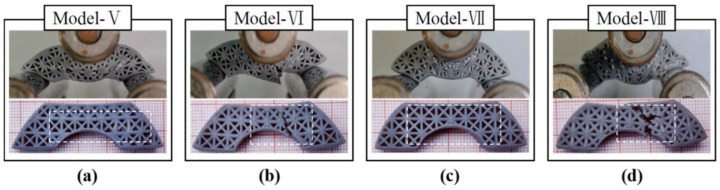
The failure modes of the topology-optimized structures: (**a**) FL–heterogeneous lattice No. 1, (**b**) FL–heterogeneous lattice No. 2, (**c**) Lattice hybrid structure with FCC strut diameter of 0.8 mm, (**d**) Lattice hybrid structure with FCC strut diameter of 1 mm.

In Figure 11, the uniform and heterogeneous lattice structure samples had different behavioral characteristics during the fracture process. As shown in Figure 11a,b, the heterogeneous lattice sample was fractured at two sides of the span, where the strut diameter was 0.6 mm. The uniform lattice structure had a brittle failure at the midpoint of the span, where the load was applied. The four lattice structures showed the failure modes of upper-surface yielding, strut fracture, and lower-surface ductile fracture. In the case of the topology-optimized models, the cracking initially occurred at the midpoint of the lower face (Figure 12). They had more deformation of the upper face than the lattices. As shown in Figure 12a,c, the buckling of the upper–lower face hardly occurred after removing the loads, leading to the maintenance of the lattice structure shape.

## 6. Conclusions

This work provides a structurally heterogeneous lattice design method suitable for the quasi-static stress state of 3D-printed parts. Lattice units inconsistently replaced the FEA mesh units with different specific rigidities corresponding to localized stress levels. Relying on the topology optimization further lightened the lattice structure under quasi-static stress after removing some parts with extremely low stress from the overall structure. As an embodiment of this design idea, the FCC lattice units with different strut diameters were employed to non-uniformly and adaptively fill a solid part under localized loading. The topological optimization was conducted on the solid part globally. Then, the topologically optimized solid and the heterogeneous lattice structure were subjected to the geometric Boolean operation. Three evaluation indicators were defined for the standardized assessment of the lattice structures for three-point bending performance evaluation. This demonstrated that the heterogeneous lattice part exhibited better comprehensive mechanical performance than the uniform lattice.

The heterogeneous lattice models tended to fracture at the minimum-strut-diameter region at the time of initial fracture. By comparison, the topology-optimized models maintained their shape after removing the load. This suggests that the proposed method could control the breaking point using different strut diameters of lattice units.

Therefore, to ensure safety, the proposed design method was expected to be introduced to the applications requiring shape maintenance and fracture location control in spite of plastic deformation or breakage. However, this design method has some limitations. Precise optimization reflecting the lattice unit within each design space was not performed, given that the heterogeneous lattice structures were created by applying various strut diameters of the lattice. Moreover, the stress concentration and weakness existed at the connection of the two lattices, due to the strut diameter difference and the discretized model used during the structure generation.

To improve the design optimization procedure’s efficiency and/or effectiveness, some further research is still needed concerning stiffness–strength analysis, lattice topology optimization, and multidisciplinary or multiphysics approaches to improve the lattice model with various materials.

## Figures and Tables

**Table 1:** Information on the designed lattice structures.

Designed Structure Case	Design Model	Relative Density, *ρ*
(I) Heterogeneous lattice No. 1	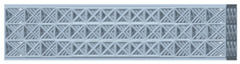	0.429
(II) Heterogeneous lattice No. 2	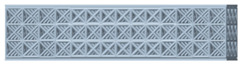	0.424
(III) Uniform lattice No. 1 (Strut diameter of 0.8 mm)	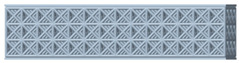	0.439
(IV) Uniform lattice No. 2 (Strut diameter of 1.0 mm)	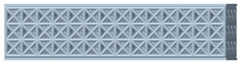	0.582
(V) FL–heterogeneous lattice No. 1	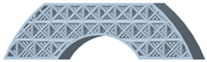	0.255
(VI) FL–heterogeneous lattice No. 2	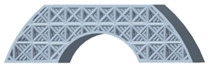	0.254
(VII) FL–uniform lattice No. 1 (Strut diameter of 0.8 mm)	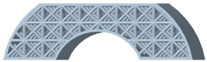	0.231
(VIII) FL–uniform lattice No. 2 (Strut diameter of 1.0 mm)	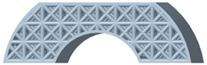	0.303

**Table 2:** Mechanical properties of the designed structures.

Designed Structure	Max. Loading, *P* [*N*]	Deflection, *δ* [mm]	Relative Flexural Rigidity $EI^{*}$	δ*	*α*	*β*	*γ*
(I) Heterogeneous lattice No. 1	727.29	5.08	0.146	1.116	0.341	2.601	0.886
(II) Heterogeneous lattice No. 2	539.65	4.74	0.103	1.02	0.243	2.456	0.598
(III) Uniform lattice No. 1 (Strut diameter of 0.8 mm)	338.71	3.03	0.103	0.666	0.234	1.518	0.355
(IV) Uniform lattice No. 2 (Strut diameter of 1.0 mm)	581.65	2.25	0.202	0.495	0.347	0.850	0.295
(V) FL–heterogeneous lattice No. 1	224.07	9.61	0.009	2.112	0.037	8.275	0.306
(VI) FL–heterogeneous lattice No. 2	231.3	11.43	0.007	2.512	0.028	9.845	0.279
(VII) FL–uniform lattice No. 1 (Strut diameter of 0.8 mm)	196.22	11.35	0.005	2.495	0.020	10.794	0.219
(VIII) FL–uniform lattice No. 2 (Strut diameter of 1.0 mm)	215.07	10.29	0.005	2.262	0.016	7.467	0.117

## Data Availability

The data presented in this study are available upon request from the corresponding author.
